# Diagnosis of inflammatory peri-implant diseases using an immunochromatographic assay for calprotectin in peri-implant crevicular fluid

**DOI:** 10.1186/s40729-021-00386-z

**Published:** 2021-10-08

**Authors:** Rie Kido, Jun-ichi Kido, Yasufumi Nishikawa, Eijiro Sakamoto, Yoritoki Tomotake, Hiromichi Yumoto

**Affiliations:** 1 Department of Periodontology and Endodontology, Institute of Biomedical Sciences, Tokushima University Graduate School, 3-18-15 Kuramoto, Tokushima, 770-8504 Japan; 2 Oral Implant Center, Tokushima University Hospital, Tokushima, Japan

**Keywords:** Calprotectin, Diagnosis, Immunochromatographic assay, Peri-implant diseases, Peri-implant crevicular fluid

## Abstract

**Background:**

The incidence rate of peri-implant diseases is increasing with implant placement. Early detection of peri-implant diseases is important to prevent and treat these diseases, and a simple and objective diagnostic method is expected. Immunochromatographic (IC) assays are used for rapid diagnostic methods for some diseases. The aim of this clinical study was to determine the amount of calprotectin, an inflammatory marker, in peri-implant crevicular fluid (PICF) using an IC chip, and estimate the possibility of this diagnostic system.

**Methods:**

Forty-six individuals with dental implants participated in a pilot study. PICF samples were collected from the peri-implant sites with or without inflammation after clinical examinations including probing depth (PD), bleeding on probing (BOP) and gingival index (GI). Calprotectin in PICF was determined by an IC chip and enzyme-linked immunosorbent assay (ELISA) for calprotectin. The density of calprotectin line on the IC chip was measured using an IC reader (IC reader value). The relationship between IC reader value and ELISA value or clinical parameters was investigated. A receiver operating characteristic (ROC) curve analysis of IC reader value of calprotectin was performed to predict inflammation in peri-implant diseases.

**Results:**

IC reader value of calprotectin was significantly correlated with its ELISA value and PD. IC reader values of calprotectin in PICF samples from periodontal sites with GI-1 and GI-2, and with BOP-positive sites were significantly higher than those of PICF samples from GI-0 sites, and BOP-negative sites, respectively. The IC reader value for calprotectin in PICF samples from inflammatory diseased sites was significantly higher than that of non-diseased sites. ROC analysis suggested that the IC reader value of PICF calprotectin was useful for predicting inflammatory peri-implant diseases.

**Conclusion:**

IC assay for PICF calprotectin may be a possible system for diagnosing the inflammatory peri-implant diseases.

## Background

The prevalence of peri-implant diseases is increasing in proportion to the increase of dental implant placement, and the mean prevalence rates of peri-implant mucositis and peri-implantitis were reported to be 43% and 22%, respectively [1]. The progression of peri-implant diseases induces an inflammation of periodontal tissues and destruction of peri-implant tissues with alveolar bone, and results in a deciduation of dental implants. The early diagnosis of peri-implant diseases is very important to protect against incidence of these diseases and to maintain dental implants. Peri-implant diseases have been examined by some clinical indicators including probing depth (PD), bleeding on probing (BOP), mucosal redness, suppuration, mobility of implant and radiographic alveolar bone loss [2–4]. The therapy for peri-implant diseases follows the standard of cumulative interceptive supportive therapy (CIST), which is classified using clinical indicators [5]. Although clinical examinations, such as PD, BOP and mobility have commonly been used for the diagnosis of peri-implant diseases, as well as periodontal diseases, their accuracy and objectivity are not necessarily sufficient because of peri-implant tissues with mucous and less attached gingiva, as well as the complex structures of dental implants and prosthetic superstructures [2, 3, 6, 7]. These problems appear to influence a wide prevalence range of peri-implant diseases; peri-implant mucositis: 19–65%, peri-implantitis: 1–47% [8, 9].

The diagnostic examination of peri-implant diseases using biomarkers in peri-implant crevicular fluid (PICF) has been studied to accurately evaluate peri-implant diseases. PICF contains some pro-inflammatory cytokines [including interleukin (IL)-1β, interleukin-6 (IL-6) and tumor necrosis factor-α (TNF-α)], proteases [including aspartate aminotransferase (AST), cathepsin K and matrix metalloproteinase-8 (MMP-8)], and bone metabolism-related proteins [including cross-linked N-telopeptide of type I collagen (NTx), receptor activator of nuclear factor-κB (RANK) and RANK ligand (RANKL)] [9, 10]. These factors have been examined as biomarkers for peri-implant diseases. Recently, we investigated the levels of calprotectin and NTx in PICF from periodontal tissues with and without peri-implant diseases [11]. Calprotectin is a heterogeneous complex of S100A8 and S100A9 and is an inflammation-related protein [12], and calprotectin level in gingival crevicular fluid (GCF) from periodontal sites with periodontal diseases was significantly higher than that of healthy sites; moreover, calprotectin level in GCF was correlated with clinical indicators of periodontal diseases, suggesting that calprotectin is a useful biomarker for periodontal diseases [13–16]. We found that calprotectin level in PICF samples from diseased sites was higher than that of healthy sites, and PICF calprotectin levels were significantly correlated with PD value and gingival index (GI) score [11]. These results suggested a possibility that calprotectin is a marker for inflammation in peri-implant diseases as well as periodontal diseases. Calprotectin in PICF was determined using a commercial enzyme-linked immunosorbent assay (ELISA) kit in our recent study, and this assay method was highly sensitive, but was complex and took approximately 4 h [11]. PICF is easily collected from crevices around dental implants using paper strips; however, the measurement of biomarkers in PICF takes a relatively long time and this measurement time is not suitable for general dental treatment.

Immunochromatographic (IC) assays have been developed for diagnosis of some infectious diseases including influenza virus, corona virus, human immunodeficiency virus, *Streptococcus pneumonia* and *Mycoplasma pneumonia* [17–21]. IC assays are useful for rapid diagnosis of infectious diseases. *Porphyromonas gingivalis* in subgingival plaque was semi-quantified using an IC device, and this device system was useful for the rapid detection of *P. gingivalis* at the dental chair-side since the assay method allows determination of *P. gingivalis* within approximately 15 min [22]. On the other hand, we developed an IC assay system to semi-determine calprotectin in GCF and investigated calprotectin level in GCF from periodontal sites with and without periodontal diseases [23]. GCF calprotectin was easily and rapidly determined using our IC assay system within approximately 15 min, and the IC reader value for GCF calprotectin significantly was correlated with PD, GI and BOP; in addition, the IC reader value of calprotectin in GCF samples from the diseased sites decreased by periodontal initial treatment [23]. These results suggested that the IC chip device for GCF calprotectin is a rapid and effective assay system for diagnosing periodontal diseases.

The aim of this study was to determine calprotectin in PICF using our IC assay system, and to evaluate the possibility of this IC assay as a simple device that estimates inflammation in peri-implant diseases.

## Methods

### Patients and clinical periodontal examinations

This clinical study was approved by the Ethics Committee of Tokushima University Hospital (No. 2719) in accordance with the Helsinki Declaration of 2013, and performed from September 2017 to April 2019 at Tokushima University Hospital. Forty-six patients (17 males and 29 females, mean ages 69.6 ± 6.9 years) with dental implants received an explanation of the present clinical study and gave written informed consent. Participants had healthy and diseased dental implants, and did not contract any systemic inflammatory diseases or take antimicrobial medicines within 3 months. The periodontal pocket depth around peri-implants was measured by three periodontists who were certified by Japanese Society of Periodontology using a peri-implant probe (Hu-Friedy, COLORVUE^®^ PROBES 3-6-8-11) by an inserting force of 20–25 g. BOP and GI of the PICF sampling sites were estimated by above three dentists. The study groups of healthy (non-inflammation) and inflammatory diseased sites of peri-implants were classified using clinical indicators including PD, BOP and GI by a specialist who was certified by Japanese Society of Oral Implantology, with reference to previous reports [4, 5, 11, 24, 25]. Healthy sites without inflammation of peri-implants were defined as periodontal sites with PD < 3, BOP-negative and GI score = 0, and inflammatory diseased sites with peri-implant diseases (peri-implant mucositis and peri-implantitis) were defined as the sites with PD ≥ 3, BOP-negative or positive and GI score ≥ 1. Clinical indicators of PD, BOP and GI were examined after the collection of PICF, and the bone loss (BL) rate of alveolar bone around peri-implants was assessed on radiographic images. GI scores were examined according to modification of the standard of Löe and Silness [26], and BL rate was determined according to a modification of Schei et al.’s method [27]. The characteristics of participants and PICF sampling sites are shown in Table 1.

**Table 1:** Characteristics of participants and PICF sampling sites

Participants	
Number (*n*)	46
Sex (male/female)	17/29
Age (years)	69.6 ± 6.9

Sampling sites	Healthy	Inflammatory	*P*-value
Number of samples	42	57	
PD (mm)	2.43 ± 0.58	5.07 ± 1.55	*P* < 0.01
Gingival index	0.00 ± 0.00	1.42 ± 0.59	*P* < 0.01
BOP-positive %	0	47.4	
Bone loss rate (%)	12.6 ± 5.0	46.8 ± 16.3	*P* < 0.01

### PICF sampling

PICF samples were collected from the peri-implant crevices using a sterile paper strip according to a modification of our previous procedure [11]. Briefly, sampling sites were isolated with cotton rolls and gently air-dried. A sterile paper strip (Shofu, Kyoto, Japan) was inserted into a peri-implant crevice and held for 30 s. Paper strips with blood and pus were not used in the present study. PICF was extracted from paper strips in 100 μL of phosphate-buffered saline (pH = 7.4) containing phenylmethylsulfonyl fluoride (0.2 μM) by centrifugation and used for the IC assay and ELISA of calprotectin.

### Calprotectin determination by IC chip and ELISA

Calprotectin amount in the extracted PICF sample was measured using an IC chip that we previously developed [23] and a Calprotectin Human ELISA kit (Hycult Biotech, PB Uden, Netherlands). This IC chip has two anti-human calprotectin antibodies (gold particle-conjugated anti-calprotectin antibody and anti-calprotectin antibody) to capture the complex of calprotectin antigen in the samples and the first antibody (Trust Medical, Hyogo, Japan). Briefly, an aliquot of extracted PICF was diluted in Tris–HCl buffer and dropped into the sample pad on the IC chip, and developed for 5 min at room temperature. The density of calprotectin line on the IC chip was measured using an IC reader (Trust Medical, Hyogo, Japan) (IC reader value). Another aliquot of the extracted PICF was diluted in Dilution Buffer from the ELISA kit and calprotectin amount in PICF sample was determined according to the instruction manual of the ELISA kit.

### Statistical analysis

Differences in PD, GI, BL rate and IC reader value between healthy and diseased groups were statistically analyzed by the Mann–Whitney *U* test. The relationships between IC reader value and ELISA value or PD were analyzed by Spearman’s rank correlation test. Differences in IC reader value among the GI-0, GI-1 and GI-2 groups, and between BOP-negative and positive groups were statistically evaluated by one-way analysis of variance (ANOVA) and Mann–Whitney *U* test, respectively. ROC curve was constructed for IC reader values of calprotectin in the healthy and inflammatory diseased groups. Data were analyzed using a statistical analysis software (Excel Analysis 2012 for Windows). *P* < 0.05 was considered significant.

## Results

### Characteristics of PICF sampling sites

Forty-two PICF samples were collected from healthy peri-implant sites without inflammation and 57 samples from the inflammatory diseased sites (Table 1). The mean PD at inflammatory sites (5.07 mm) was significantly deeper than that of healthy sites (2.43 mm). The GI score of inflammatory sites was 1.42, which was significantly higher than that of healthy sites (GI-0), and BOP-positive percentage at inflammatory sites was 47.4%. Furthermore, the mean BL rate of inflammatory sites was 46.8%, which was significantly higher than that of healthy sites (12.6%) and was approximately 3.7-fold.

### Correlation between IC reader value and ELISA value of PICF calprotectin or PD

**Fig. 1:**
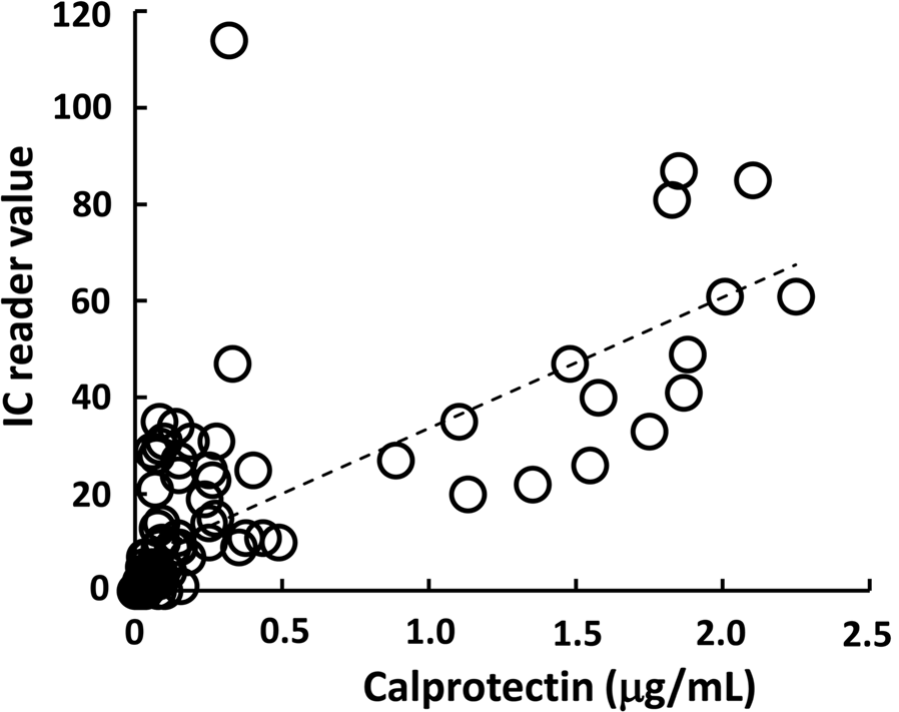
Correlation between IC reader value and ELISA value of PICF calprotectin amount. Calprotectin amount in PICF samples (*n* = 99) were determined by IC assay and ELISA. Correlation coefficient was 0.843 (*P* < 0.001)

**Fig. 2:**
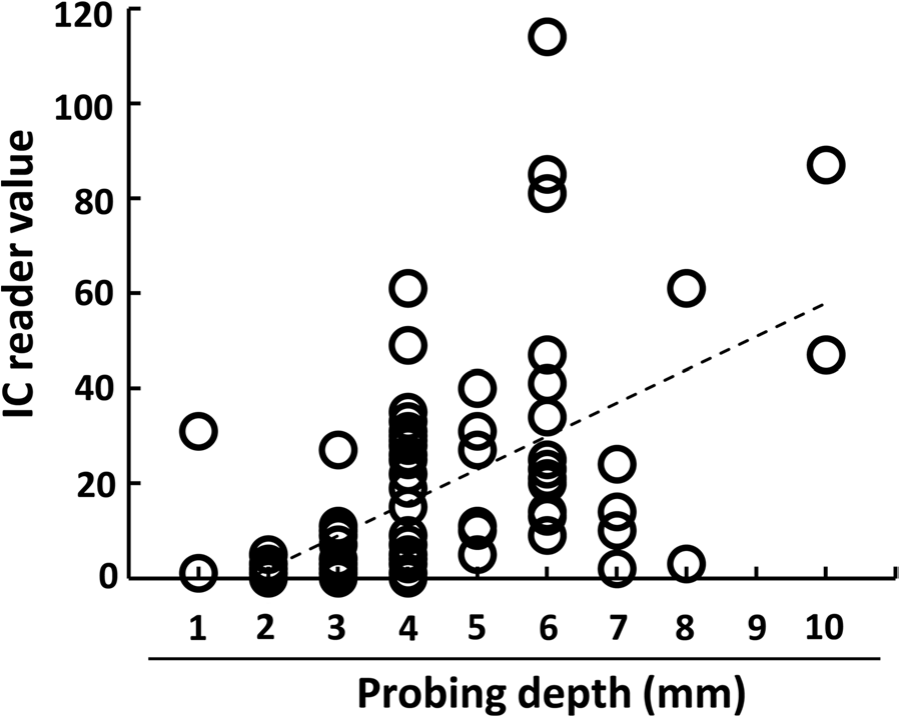
Correlation between IC reader value of PICF calprotectin amount and PD. The correlation between IC reader value of calprotectin and PD was evaluated in 99 PICF samples and sites (*ρ* = 0.678, *P* < 0.001)

IC reader value for PICF calprotectin ranged from 0 to 87, and ELISA value (calprotectin concentration) ranged from 0 to 2.25 μg/mL, and IC reader value was positively correlated with ELISA value (*ρ* = 0.843, *P* < 0.001, Fig. 1). PD ranged from 0 to 10 mm, and IC reader value of PICF calprotectin was also significantly correlated with PD (*ρ* = 0.678, *P* < 0.001, Fig. 2).

### Relationship between IC reader value of calprotectin and clinical indicators

**Fig. 3:**
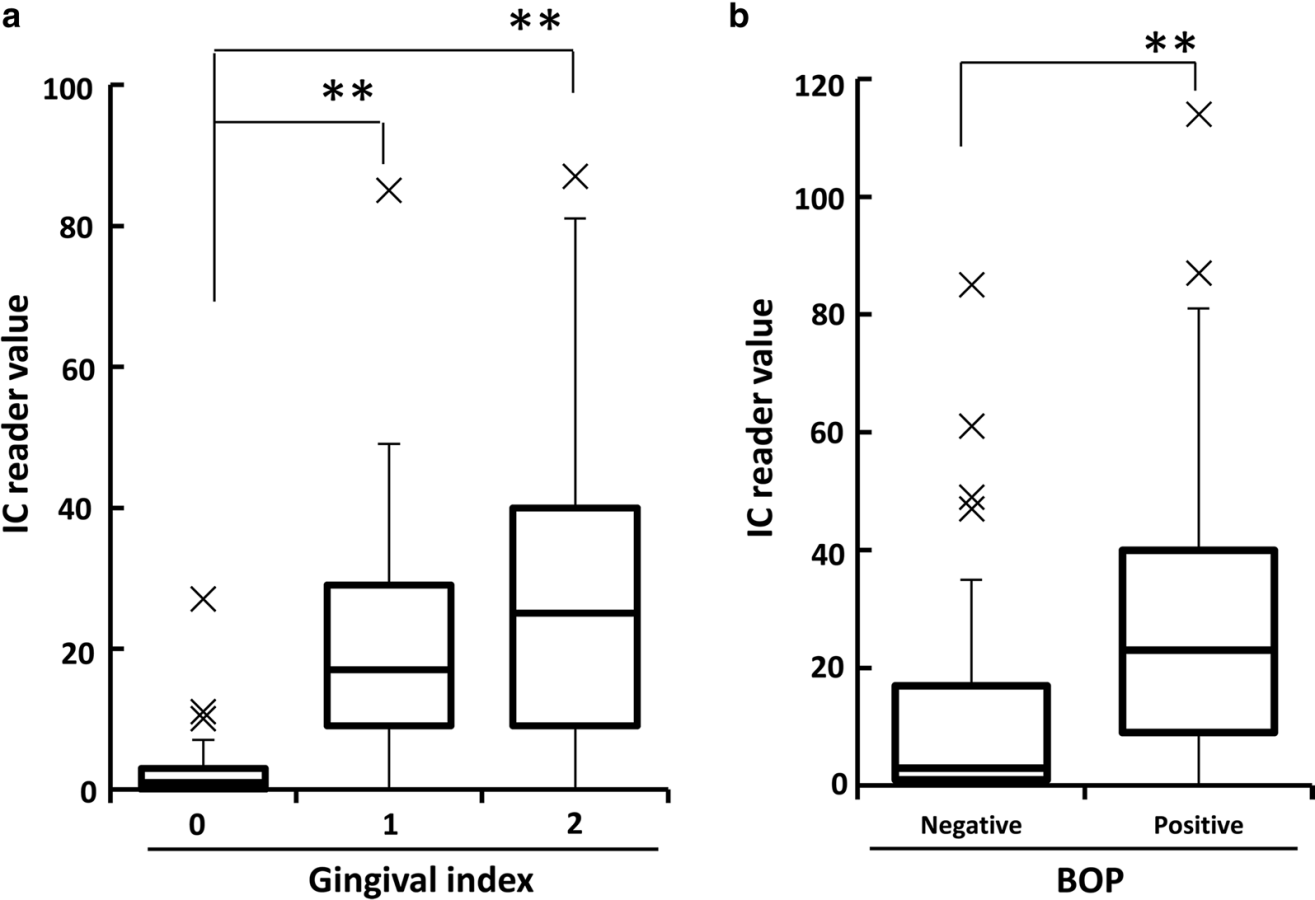
Relationship between IC reader value of PICF calprotectin amount and GI score or BOP. **a** The relationship between IC reader value of calprotectin amount in PICF samples from the sites with GI-0 (*n* = 42), GI-1 (*n* = 30) and GI-2 (*n* = 27), and GI scores were statistically analyzed. **G-1 and G-2 groups versus G-0 group, *P* < 0.01. **b** The IC reader value of calprotectin amount in PICF samples from the BOP-negative (*n* = 72) and BOP-positive (*n* = 27) sites was compared. **BOP-positive versus BOP-negative, *P* < 0.01

The mean IC reader values of GI-0, GI-1 and GI-2 groups were 2.5, 20.6 and 30.7, respectively, and their median IC reader values were 1, 17 and 25 (Fig. 3a). IC reader values of PICF samples from periodontal sites with GI-1 and GI-2 were significantly higher than that of GI-0 (*P* < 0.01). However, there was no significant difference in IC reader values between GI-1 and GI-2 (Fig. 3a). The mean IC reader values of PICF calprotectin from BOP-negative and positive sites were 11.6 and 30.7, respectively, and the median IC reader values were 3 and 23, and the IC reader value of the BOP-positive group was significantly higher than that of the BOP-negative group (*P* < 0.01, Fig. 3b).

### Comparison of IC reader value between PICF samples from healthy and inflammatory sites, and ROC analysis of PICF calprotectin

**Fig. 4:**
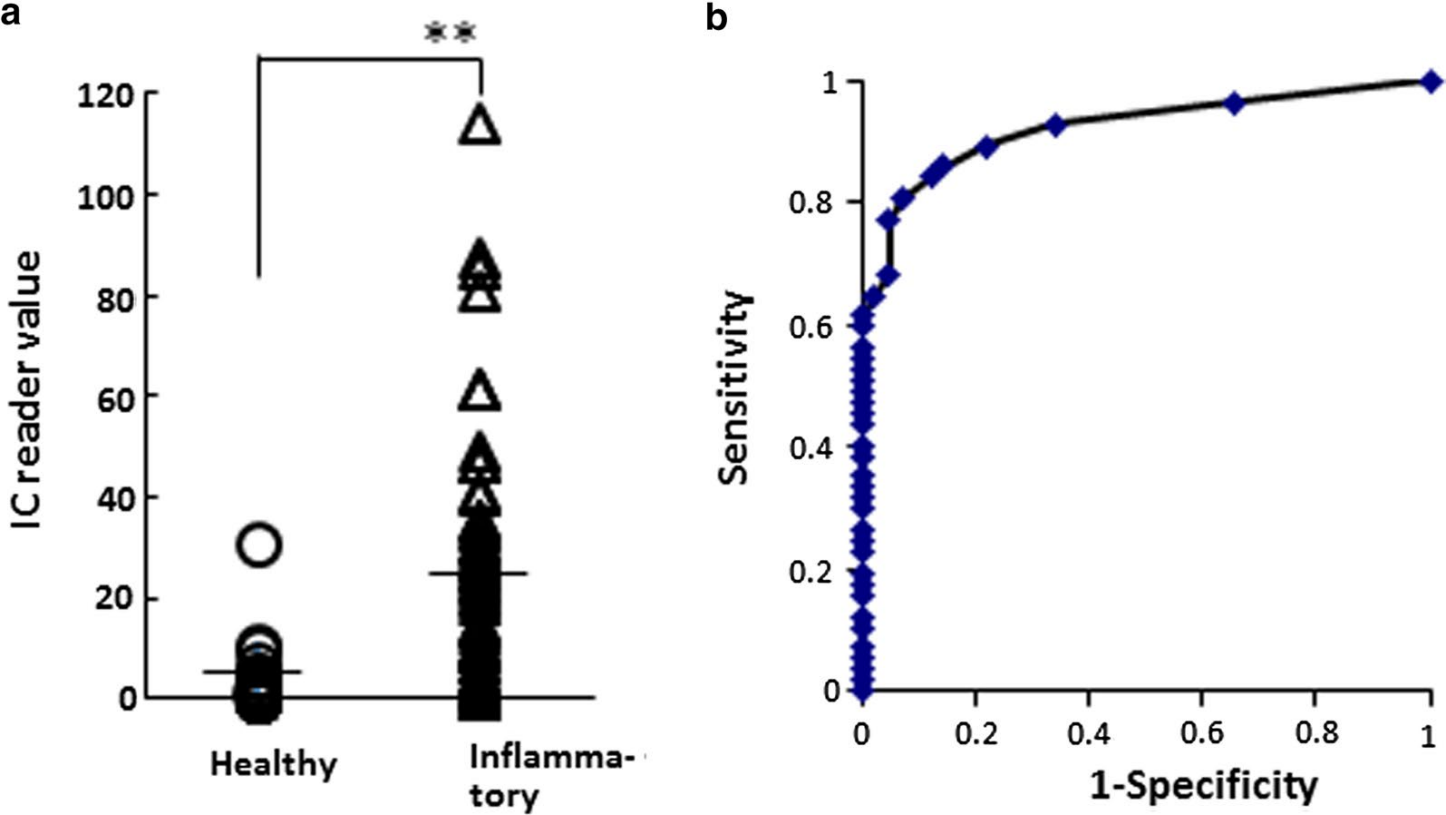
ROS analysis of PICF calprotectin amount determined using an IC reader to predict peri-implant diseases. **a** The IC reader value of calprotectin in PICF samples from the inflammatory diseased sites (*n* = 57) and healthy sites (*n* = 42) was compared. Horizontal bars show the mean values of the inflammatory and healthy groups. **P < 0.01. **b** The IC reader value of PICF calprotectin amount was subjected to ROC curve analysis. AUC value for the IC reader value of calprotectin amount was 0.908 (95% CI 0.847–0.969, *P* < 0.001). When the cutoff value was 5.0, the sensitivity and specificity were 84.2% and 85.7%, respectively

After the inflammatory and healthy groups were defined, the mean IC reader values of inflammatory and healthy groups were calculated as 2.5 and 25.7, respectively (Fig. 4a). The mean IC reader value in the inflammatory group was significantly higher than that of the healthy group (*P* < 0.01). ROC curve for the IC reader value of PICF calprotectin was plotted to predict peri-implant diseases. The area under the ROC curve (AUC) was 0.908, the 95% confidence interval (95% CI) was 0.847 to 0.969 and the *P* value was < 0.001. The cutoff value of IC reader value was 5.0, and its sensitivity and specificity were 84.2% and 85.7%, respectively (Fig. 4b).

## Discussion

Some studies to diagnose peri-implant diseases using biomarkers in PICF were performed in association with an increase of dental implant treatments [9, 10]. The examination using PICF biomarkers makes an objective diagnosis of peri-implant diseases possible. The levels of IL-1β, IL-6, TNF-α and prostaglandin E2 (PGE_2_) in PICF from peri-implant diseases were significantly higher than those of healthy sites [28–31], whereas no difference between diseased peri-implant and healthy implant groups in IL-1β, and IL-6 and PGE_2_ levels were reported [32, 33]. The reason behind these contrasting results is not clear. We speculate that the differences of definition of PICF sampling sites and its methods, or stability of those cytokines and PGE_2_ after sample collecting might affect opposite results. Calprotectin is known to be heat stable [34] and has been studied as a diagnostic marker for periodontal diseases [13, 14, 16, 35]. Friedmann et al*.* [36] detected calprotectin in crevicular fluid around peri-implants and its level was similar to the GCF calprotectin level. We recently reported that calprotectin level in PICF from peri-implantitis sites was higher than that of healthy sites [11]. The significant difference in calprotectin level (*P* < 0.001) between peri-implantitis and healthy groups was more marked than those of IL-1β (*P* = 0.002 or *P* = 0.02) and IL-6 (*P* = 0.049 or *P* = 0.02) [30, 37], suggesting that calprotectin in PICF is a possible marker for diagnosing peri-implant diseases with inflammation.

Monje et al*.* [4] diagnosed peri-implant diseases by generalized estimating equations of multilevel logistic regression of clinical parameters including PD, BOP, mucosal redness, suppuration and plaque index. When peri-implantitis was diagnosed by their estimating method with clinical parameters, the sensitivity and specificity were 52.5% and 92.1%, respectively, and the AUC value in the ROC curve was 0.81 (95% CI 0.78–0.84). In the present study, the PICF calprotectin assay using our IC chip had a sensitivity of 84.2% and specificity of 85.7%. The sensitivity of our assay method (84.2%) was much higher than that of Monje’s method (52.5%) using clinical parameters [4]. Furthermore, the AUC value was 0.908 (95% CI 0.847–0.969) in the ROC curve of our assay, and showed a higher level compared to the values by clinical parameters (AUC = 0.81, 95% CI 0.78–0.84). These results suggest that the semi-determination of PICF calprotectin using our IC chip is a possible method for diagnosing inflammatory peri-implant diseases.

In the diagnostic assay using ELISA of PICF calprotectin, a higher sensitivity (92.5%) and specificity (90.9%) was demonstrated, and the AUC value was 0.964 [11]. Although these values were higher than those of the IC chip assay, it took approximately 4 h to measure calprotectin protein level by ELISA, and this time length is not suitable for dental treatment in the dental office. The IC assay used in the present study allows a diagnosis of inflammation in peri-implant diseases possible within a very short time (approximately 15 min). The IC reader value was well correlated to calprotectin amount in the ELISA and showed a high correlation coefficient (*ρ* = 0.843, *P* < 0.001), suggesting that the IC assay for PICF calprotectin may contribute to the selection of reasonable treatments as a point-of-care test of peri-implant diseases.

There are a few studies in which the relationship between biomarker level in PICF and clinical parameters was investigated. The levels of TNF-α and AST activity in PICF were significantly correlated with PD, with a correlation coefficient of 0.3038 and 0.55, respectively [38, 39]. PICF calprotectin amount assayed by an IC chip was also significantly correlated with PD value (*ρ* = 0.678, *P* < 0.001) in the present study. This correlation was similar to that between PICF calprotectin amount determined by ELISA and PD value (*ρ* = 0.709, *P* < 0.001) [11], and was stronger than the correlation between GCF calprotectin amount and PD in a diagnosis of periodontitis using the same IC chip (*ρ* = 0.557, *P* < 0.001) [23], and between the levels of TNF-α or AST activity and PD [38, 39]. When the relationship between PICF calprotectin amount and GI score were investigated in the assays using IC chip and ELISA, this relationship showed a similar pattern in which calprotectin level in PICF samples from the peri-implant sites with GI score of 1 and 2 were significantly higher than that of healthy (GI = 0) sites, although there was no significant difference between GI = 1 and GI = 2 groups. The IC reader value for calprotectin in PICF samples from BOP-positive sites was significantly higher than that of BOP-negative sites. These results suggest that calprotectin in PICF may detect the early stage of inflammation in peri-implant diseases and its level shows more clear correlation with clinical parameters than that of other biomarkers.

In the ROC curve for predicting peri-implant diseases using an IC chip, the AUC for the IC reader value of PICF calprotectin was relatively high (0.908, 95% CI 0.847–0.969), and the sensitivity and specificity were 84.2% and 85.7%, respectively. The AUC value, sensitivity and specificity in the assay using an IC chip were slightly lower than those from ELISA determination, in which the AUC, sensitivity and specificity were 0.964, 92.5% and 90.9%, respectively [11]. However, these values were high enough to predict peri-implant diseases in comparison with the AUC value (0.735) of TNF-α in PICF [37], as well as the sensitivity (0.81) and specificity (0.74) for AST activity [39] and sensitivity (0.73) and specificity (0.73) for IL-1β [40]. When periodontal diseases were diagnosed by determining GCF calprotectin using the same IC chip, the AUC value in the ROC assay was 0.826 (95% CI 0.772 to 0.879, *P* < 0.001), and the sensitivity and specificity for predicting periodontal diseases were 73% and 82%, respectively [23]. The AUC value, sensitivity and specificity in ROC assay to predict peri-implant diseases were almost similar to those values of diagnosis of periodontal diseases. These results suggest a possibility that the semi-determination of calprotectin in PICF and GCF using an IC chip system as a suitable alternative to ELISA, is effective for diagnosing inflammatory peri-implant diseases as well as periodontal diseases.

## Conclusions

The present study suggests a possibility that IC assay for PICF calprotectin is useful as a point-of-care test for diagnosing inflammatory peri-implant diseases.

## Data Availability

All data generated in this study are included in this article.

## Abbreviations

AST: Aspartate aminotransferase
AUC: Area under the ROC curve
BOP: Bleeding on probing
CIST: Cumulative interceptive supportive therapy
ELISA: Enzyme-linked immunosorbent assay
GCF: Gingival crevicular fluid
GI: Gingival index
IC: Immunochromatographic
IL-1β: Interleukin-1β
IL-6: Interleukin-6
MMP-8: Matrix metalloproteinase-8
NTx: Cross-linked N-telopeptide of type I collagen
PICF: Peri-implant crevicular fluid
PD: Probing depth
PGE_2_: Prostaglandin E_2_
RANK: Receptor activator of nuclear factor-κB
RANKL: RANK ligand
ROC: Receiver operating characteristic
TNF-α: Tumor necrosis factor-α
